# Early childhood caries risk prediction using machine learning approaches in Bangladesh

**DOI:** 10.1186/s12903-025-05419-2

**Published:** 2025-01-08

**Authors:** Fardous Hasan, Maha El Tantawi, Farzana Haque, Moréniké Oluwátóyìn Foláyan, Jorma I. Virtanen

**Affiliations:** 1 Department of Clinical Dentistry, Faculty of Medicine, University of Bergen, Bergen, Norway; 2 Department of Pediatric Dentistry and Dental Public Health, Faculty of Dentistry, Alexandria University, Alexandria, Egypt; 3 Early Childhood Caries Advocacy Group, University of Manitoba, Winnipeg, Canada; 4 Department of Child Dental Health, Obafemi Awolowo University, 22005 Ile-Ife, Nigeria; 5 Oral Health Initiative, Nigerian Institute of Medical Research, Yaba, Lagos, Lagos State 100001 Nigeria; 6 Institute of Dentistry, University of Turku, Turku, Finland

**Keywords:** Dental caries, Children, Artificial intelligence, Machine learning, Risk

## Abstract

**Background:**

In the last years, artificial intelligence (AI) has contributed to improving healthcare including dentistry. The objective of this study was to develop a machine learning (ML) model for early childhood caries (ECC) prediction by identifying crucial health behaviours within mother-child pairs.

**Methods:**

For the analysis, we utilized a representative sample of 724 mothers with children under six years in Bangladesh. The study utilized both clinical and survey data. ECC was assessed using ICDAS II criteria in the clinical examinations. Recursive Feature Elimination (RFE) and Random Forest (RF) was applied to identify the optimal subsets of features. Random forest classifier (RFC), extreme gradient boosting (XGBoost), support vector machine (SVM), adaptive boosting (AdaBoost), and multi-layer perceptron (MLP) models were used to identify the best fitted model as the predictor of ECC. SHAP and MDG-MDA plots were visualized for model interpretability and identify significant predictors.

**Results:**

The RFC model identified 10 features as the most relevant for ECC prediction obtained by RFE feature selection method. The features were: plaque score, age of child, mother’s education, number of siblings, age of mother, consumption of sweet, tooth cleaning tools, child’s tooth brushing frequency, helping child brushing, and use of F-toothpaste. The final ML model achieved an AUC-ROC score (0.77), accuracy (0.72), sensitivity (0.80) and F1 score (0.73) in the test set. Of the prediction model, dental plaque was the strongest predictor of ECC (MDG: 0.08, MDA: 0.10).

**Conclusions:**

Our final ML model, integrating 10 key features, has the potential to predict ECC effectively in children under five years. Additional research is needed for validation and optimization across various groups.

## Introduction

Early Childhood Caries (ECC), referring to decayed, missing, or filled primary teeth in children younger than 72 months, affected 514 million children in 2022 [1]. Its estimates indicate a prevalence of 24% and 57% in 0–2 and 3–5-year-old children [2] with an overall global estimate of 48% [3, 4]. Although there are few studies on ECC from Africa and South Asia - continents that host the majority of low- and middle-income countries [5, 6] - the few studies indicate that the burden of ECC is high due to the high rate of population growth, suboptimal oral hygiene, and limited access to care [6].

Left untreated, ECC develops complications associated with dental discomfort and pain, oral infections, sleep disturbance and poor quality of life, among other complications [7]. Other long-term consequences include a high risk for dental caries in the permanent dentition, poor oral health outcomes in adulthood, and detrimental impacts on the physical and psychological well-being [8, 9]. Thus, preventive care and early prediction of ECC are crucial in mitigating its negative impact.

Various approaches have been developed to predict the risk of dental caries by identifying individuals at high risk of developing dental caries, allowing for targeted preventive measures and personalized treatment plans. These approaches utilize a combination of clinical, behavioral, and biological dentists to assess a patient’s susceptibility. Clinically, dentists assess for the presence of existing carious lesions, plaque accumulation, and previous dental history. Factors like tooth morphology, fluoride exposure, and salivary flow are also taken into account [10]. Behavioural dental caries risk indicators for children include high sugar intake, infrequent brushing, and irregular professional cleanings [11]. Biological indicators are bacterial load, such as *Streptococcus mutans* and *Lactobacilli* levels, saliva’s buffering capacity and saliva flow rate [12]. Formalized caries risk assessment models integrate clinical, behavioral, and biological data to produce a comprehensive evaluation of an individual’s risk profile. Such formalised tools include Caries Management by Risk Assessment (CAMBRA) [13] and Cariogram [14].

Despite these methods, predicting dental caries remains complex due to the multifactorial nature of the disease. Caries development is influenced by a dynamic interplay of genetic, environmental, and lifestyle factors, making it challenging to pinpoint risk with absolute accuracy [15]. For infants, toddlers and pre-school children, the risk of ECC is further influenced by the maternal education and income levels, and parents’ health behaviours [5, 16, 17]. Access to healthcare services is also associated with the risk for ECC [18, 19]. However, continued refinement of risk assessment tools, combined with a tailored preventive approach, holds promise for improving the early detection and prevention of caries.

Artificial intelligence (AI) can help with the refinement of risk assessment as it enables more precise predictions and optimization of public health interventions [20, 21]. It can improve the detection, prevention, and management of root caries [22], caries in second molars due to impacted third molars [23] and untreated caries in adolescents [24]. Novel predictive ML models may identify key factors influencing dental health, and thereby promoting health and preventing ECC.

Machine learning (ML) is a branch of AI whose algorithms can be used to analyse socioeconomic data to predict healthcare needs, disease outbreaks and identify at-risk populations [25, 26]. Its use for the prediction of ECC is still in its early stages with an earlier study using XGBoost, random forest, and LightGBM on Korean 1 to 5-year olds [27]. In addition, Karhade et al. created an automated machine learning (AutoML) model to classify ECC in children aged 3 to 5 years from North Carolina, highlighting its potential for ECC screening [28]. Thus, the use of ML to automate the diagnosis of ECC, especially in low resource setting were the burden of ECC seems to outweigh the human resource capacity of diagnose and manage the high disease burden.

Bangladesh is a lower-middle-income country [29] with a low dentist to population ratio [30]. The automation of some patient care processes may be of value. No study has been conducted in Bangladesh to assess the performance of ML to predict ECC. Context specific factors are expected to affect the variables selected for inclusion in ML models and their impact on the prediction. The goal of this study was to develop a ML model for ECC prediction in Bangladesh taking into consideration some of the context specific factors that influence the predictive ability of the ML. Specifically, the goal of the study was to identify critical health behaviours of mother-child pairs that can be used to build a high-performance model capable of accurately predicting the risk of ECC in children in Bangladesh. The research question of this study was: What are the ECC risk indicators that ML can identify?

## Methods

### Study design and study participants

This was a secondary data analysis of primary data collected from 724 mother-child dyads in Trishal, Bangladesh in 2021–2022 to determine the risk indicators for ECC in the population [17, 31]. The children’s ages ranged from 1 to 5 years.

ECC was assessed using ICDAS II criteria [32] by a calibrated dentist which classifies dental caries on a scale from 0 to 6, to conduct the clinical examinations. Dental plaque was examined on the labial surfaces of the upper central incisors and classified as one of the following: ‘No visible plaque’, ‘Plaque present only at the gingival margin’, or ‘Abundant plaque covering more than the gingival margin’.

In addition, data on the age of the mother and child, the total number of children in the family, mothers’ knowledge, attitudes, and behaviour regarding their child’s oral health were collected [17, 31]. Mothers’ knowledge was assessed through statements like “Importance of baby teeth,” “Use of fluoride toothpaste,” “No need for dentist unless problems,” “Avoid sharing spoon,” and “Monthly teeth checking”, and the responses were categorized as either “correct” or “don’t know/incorrect”. Additionally, eight statements reflecting mothers’ attitudes towards parental intentions and perceptions of their child’s tooth brushing habits and daily sugar consumption were grouped into “Agree,” “Disagree,” or “Don’t Know”. Mothers’ behaviours, such as use of dental services, fluoridated toothpaste, and need for adult assistance during brushing, were recorded as “Yes” or “No”. Brushing frequency for both mother and child was categorized from “Twice a day” to “Never”. Methods for cleaning the child’s teeth were documented as “Toothbrush,” “Toothpaste,” “Washcloth/Gauze,” or “Water”. Data was collected using a validated questionnaire adapted to the local context [33, 34]. The Cronbach’s alpha of the tool was 0.78.

### Data analysis

To develop the ML model, the data set went through preprocessing steps. The child’s age was discretized into tertiles based on the 33^rd^ and 67^th^ percentiles, resulting in three groups: 12–31 months, 32–46 months, and 47–59 months. The mother’s age was categorized as younger (≤ 24 years) or older (> 24 years). The mother’s level of education was categorized as basic, primary, secondary, and tertiary, and the total number of children in the family was grouped into 1, 2, and > 2. ECC was categorized as 0 (ICDAS = 0) and 1 (ICDAS = 1–6). Plaque scores were classified as 0 for the absence of plaque and 1 for the presence of plaque on any surface of the tooth.

Correlation heatmap was used among predictor features of the dataset to identify correlated pairs and omit features with a correlation coefficient exceeding 0.7 to avoid multicollinearity in the feature set.

In this study, we used two distinct feature selection methods to get the most relevant subset of features (variables) individually for each method. Firstly, we used recursive feature elimination (RFE), which eliminates the redundant variables that reduce the performance of the model [35]. In this method, weighted score was used to evaluate and rank the importance of features. For this, we used a combination of accuracy and F1 score with weights of 0.7 and 0.3, respectively. This method revealed a set of 10 features: plaque score, age of child, mother’s education, number of siblings, age of mother, consumption of sweet, tooth cleaning tools, child’s tooth brushing frequency, helping child brushing, and use of fluoridated toothpaste.

Secondly, we used the random forest (RF) method for feature selection where a threshold value of 0.023 was used to obtain a subset of features with higher performance metrics than the RFE feature selection method [36]. The RF method revealed a set of 14 features: the same 10 features obtained by the RFE method, and four other features: How often do you brush your own teeth? It is best to use toothpaste with fluoride when brushing a child’s teeth; I don’t know how to brush my child’s teeth properly; If our child doesn’t want to brush his teeth every day, we don’t feel we should make them.

After feature selection, supervised ML models were developed using RF classifier (RFC), extreme gradient boosting (XGBoost), support vector machine (SVM), adaptive boosting (AdaBoost), and multi-layer perceptron (MLP). The dataset was partitioned into three sets: a training set to fit the model, constituting 70% of the data (506 observations), a validation set to evaluate and fine tune the model comprising 15% of the data (109 observations), and a test set for the evaluation of the final model, representing 15% of the data (109 observations).

**Fig. 1:**
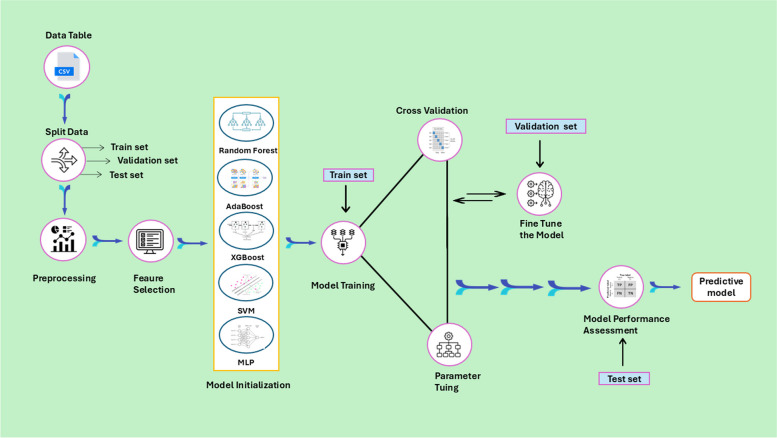
The flow chart of the machine learning modelling

A Bayesian encoding approach i.e., target encoding, was employed so that each value of the categorical feature is associated with the conditional mean of the target variable (the posterior probability of the target) given the specific value of the feature [37]. The ML models were fine-tuned by grid search cross validation (GridSearchCV) to identify the optimal set of hyperparameters [38]. In the tuning process, K-fold cross-validation (K-fold CV) with 5 folds was incorporated [39]. In each iteration, the training set was divided into five subsets, with four serving as the training set and one as the validation set. This process was repeated until each of the subsets had been used as a validation set. The entire loop iterates 10 times to robustly assess the model’s performance. Afterward, to ensure a minimum sensitivity of 80%, a threshold was determined by maximizing the sum of sensitivity, and specificity on the training set [40, 41]. Thereafter, the determined threshold was applied to the validation and test sets. The process of applying ML within Knowledge Discovery in Databases (KDD) is illustrated in Fig. 1.

**Fig. 2:**
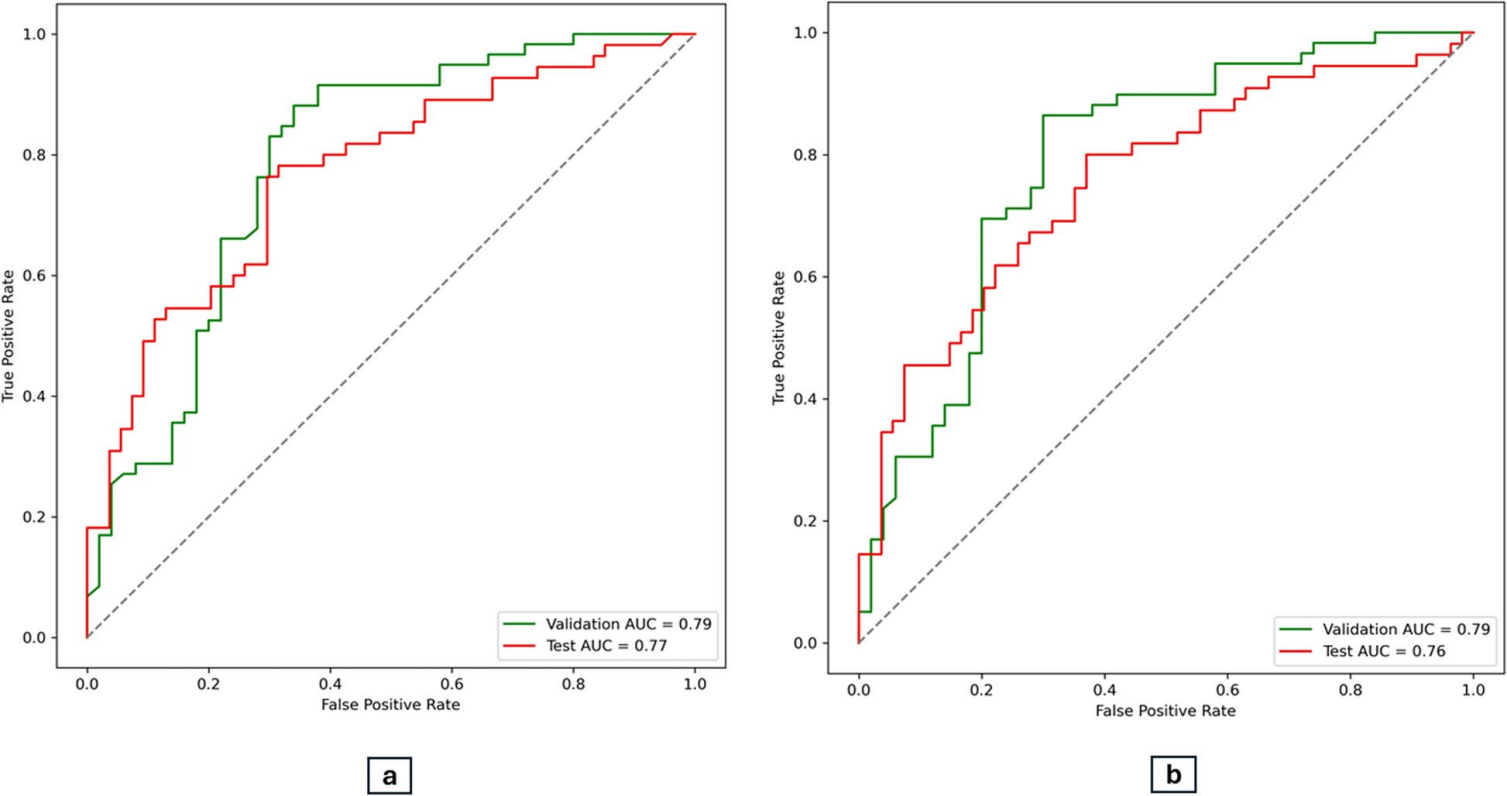
ROC curves showing the performance of the ML model using (**a**) RFE and (**b**) RF feature selection methods for both the test and validation sets, with corresponding AUC-ROC values. The ROC curve is plotted with the True Positive Rate (sensitivity) on the y-axis and the False Positive Rate (1-specificity) on the x-axis

We generated receiver operating characteristic (ROC) curves and computed the area under the ROC curve (AUC-ROC) for validation and test set. These AUC-ROC curves were depicted in a multi-plot figure, featuring corresponding AUC values in the legend. This graphical representation offered insights into the discrimination capacity of multiple ML models across the two feature selection methods. A comprehensive performance overview was provided by incorporating the AUC-ROC, accuracy, sensitivity, specificity, and F1 score for each ML model. The analysis of these performance metrics facilitated a thorough evaluation, revealing the model’s strengths. Based on these metrics (Table 1; Fig. 2 (a, b)), the best model was identified. Using this final model, we explain and interpret the findings.

**Table 1:** Descriptive characteristics of demographics, and oral health-related predictor variables (*n* = 724)

Characteristics	Values	n (%)
Age of mother (years)	17 - 24	275 (37.98)
	25 - 55	449 (62.02)
Age of child (months)	12 - 31	195 (26.93)
	32 - 46	287 (39.64)
	47 – 59	242 (33.43)
Number of siblings	1	223 (30.80)
	2	345 (47.65)
	>2	156 (21.55)
Mother’s education	Basic	50 (6.91)
	Primary	323 (44.61)
	Secondary	246 (33.98)
	Tertiary	105 (14.50)
Baby teeth are as important as the adult teeth	Correct	654 (90.33)
	Don’t know/ Incorrect	70 (9.67)
It is best to use toothpaste with fluoride when brushing a child’s teeth	Correct	67 (9.25)
	Don’t know/ Incorrect	657 (90.75)
There’s no need to go to the dentist unless children have problem with their teeth	Correct	29 (4.01)
	Don’t know/ Incorrect	695 (95.99)
Mother should avoid sharing spoon	Correct	670 (92.54)
	Don’t know/ Incorrect	54 (7.46)
Parents checking their child’s teeth every month for changes or spots	Correct	670 (92.54)
	Don’t know/ Incorrect	54 (7.46)
There is no need to go to dentist unless children have problem in their teeth	Correct	29 (4.01)
	Don’t know/ Incorrect	695 (95.99)
We intend brushing our child’s teeth for him ⁄ her twice a day	Agree	714 (98.62)
	Don’t Know/ Disagree	10 (1.38)
My family feel it was important to help in brushing our child’s teeth twice a day	Agree	716 (98.90)
	Don’t Know/ Disagree	8 (1.10)
We feel able to brush our child’s teeth for him ⁄ her	Agree	687 (94.89)
	Don’t Know/ Disagree	37 (5.11)
I don’t know how to brush my child’s teeth properly	Agree	125 (17.27)
	Don’t Know/ Disagree	599 (82.73)
We don’t have time to help brush our child’s teeth twice a day	Agree	174 (24.03)
	Don’t Know/ Disagree	550 (75.97)
It is worthwhile to give our child sweets ⁄ biscuits to behave well	Agree	412 (56.91)
	Don’t Know/ Disagree	312 (43.09)
In our family, it is fair to give sweets to our child every day	Agree	419 (57.87)
	Don’t Know/ Disagree	305 (42.13)
If our child doesn’t want to brush his teeth every day, we don’t feel we should make them	Agree	282 (38.95)
	Don’t Know/ Disagree	442 (61.05)
During the past year, has your child been to the dentist or dental clinic for a routine check-up or cleaning?	Yes	21 (2.90)
	No	703 (97.10)
Has your child ever had his/her teeth checked by a dentist or other care provider?	Yes	26 (3.59)
	No	698 (96.41)
Do you or another adult help your child brush his or her teeth?	Yes	210 (29.01)
	No	514 (70.99)
When your child’s teeth are brushed, is fluoride toothpaste usually used?	Yes	339 (46.82)
	No	385 (53.18)
How often do you brush your own teeth?	Twice a day	233 (32.18)
	Once a day	491 (67.82)
	Sometimes	0
	Never	0
How often are your child's teeth brushed? (twice or more each day)	Twice a day	161 (22.24)
	Once a day	483 (66.71)
	Sometimes	46 (6.35)
	Never	34 (4.70)
How do you clean your child’s teeth?	Toothbrush	547 (75.55)
	Toothpaste	52 (7.18)
	Washed cloth / gauge	32 (4.42)
	Water	93 (12.85)

A bar plot was generated to visualize the feature importance rankings based on mean decrease gini (MDG) and mean decrease accuracy (MDA) for the selected features. The horizontal bars indicated the magnitude of importance, with higher bars corresponding to more influential features.

SHAP (Shapley Additive Explanations) plot was used to make the ML model more interpretable and explainable [42]. Through the SHAP beeswarm plot, global interpretability and transparency were achieved by illustrating the impact of features as well as their directionality across the entire dataset. Features with positive SHAP values positively impact the predicted outcome, and vice versa. The magnitude indicates the strength of the effect. The colour of each point on the graph represents the value of the corresponding feature, with red indicating high values and blue indicating low values. Each point represents a row of data from the original dataset. SHAP shows the importance of each feature on the prediction of the model. In our study, we used open-source Python version 3.11.5 packaged by Anaconda. The analysis utilized several Python libraries included Pandas, NumPy, Scikit-learn, Matplotlib, and xgboost.

## Results

As shown in Tables 1 and 449 (62.0%) mothers were 25–55 years old, 156 (21.6%) had more than two children, 323 (44.6%) had primary education, 419 (57.9%) agreed that it is fair to give sweets to our child every day, and 514 (71.0%) did not help their child during brushing.

There were 195 (26.9%) children between 12-31 months, 287 (39.6%) between 32-46 months, and 242 (33.4%) between 47-59 months. In addition, 385 (53.2%) children did not use fluoridated toothpaste, 547 (75.6%) had mothers clean their teeth with toothbrushes and 483 (66.7%) brushed their teeth once a day. Furthermore, 375 (51.8%) of the children had ECC, and 475 (65.6%) children had dental plaque on any surface of one or more teeth.

Table 2 shows that Random Forest and XGBoost were the top performing models. The RFC, employing both RFE and RF feature selection methods, outperformed XGBoost. Within the RFC method, the RFE feature selection method demonstrated higher performance, achieving AUC-ROC of 0.77 compared to 0.76, and accuracy of 0.72 compared to 0.71 for the RF feature selection method, with both methods yielding comparable F1-scores of 0.73. Overall, the RFC utilizing RFE feature selection showed the best performance in predicting outcomes.

**Table 2:** Performance metrics of the test set for different machine learning models using the RFE feature selection (10 features) and RF feature selection (14 features): accuracy, sensitivity, specificity, F1-score

Model	FST^a^	Threshold	Accuracy	Sensitivity	Specificity	F1-Score
Random Forest	RFE	0.51	0.72	0.80	0.61	0.73
	RF	0.49	0.71	0.80	0.61	0.73
XGBoost	RFE	0.51	0.70	0.80	0.59	0.73
	RF	0.52	0.69	0.78	0.59	0.72
SVM	RFE	0.67	0.68	0.80	0.56	0.72
	RF	0.67	0.67	0.80	0.54	0.71
MLP	RFE	0.52	0.65	0.78	0.52	0.69
	RF	0.52	0.67	0.78	0.56	0.71
AdaBoost	RFE	0.46	0.67	0.82	0.52	0.71
	RF	0.46	0.68	0.82	0.54	0.72

^a^*FST *Feature selection technique

Figure 2a and b illustrate the AUC-ROC score for the RFC combined with RFE and RF feature selection respectively. The AUC-ROC score for the RFC method with RFE feature selection on the test set was 0.77. The final model achieved an accuracy of 0.72, sensitivity of 0.80, and an F1-score of 0.73 (Table 2) underscoring the model’s balance between precision and recall, and highlighting its acceptable capability to accurately identify true positive cases.

**Fig. 3:**
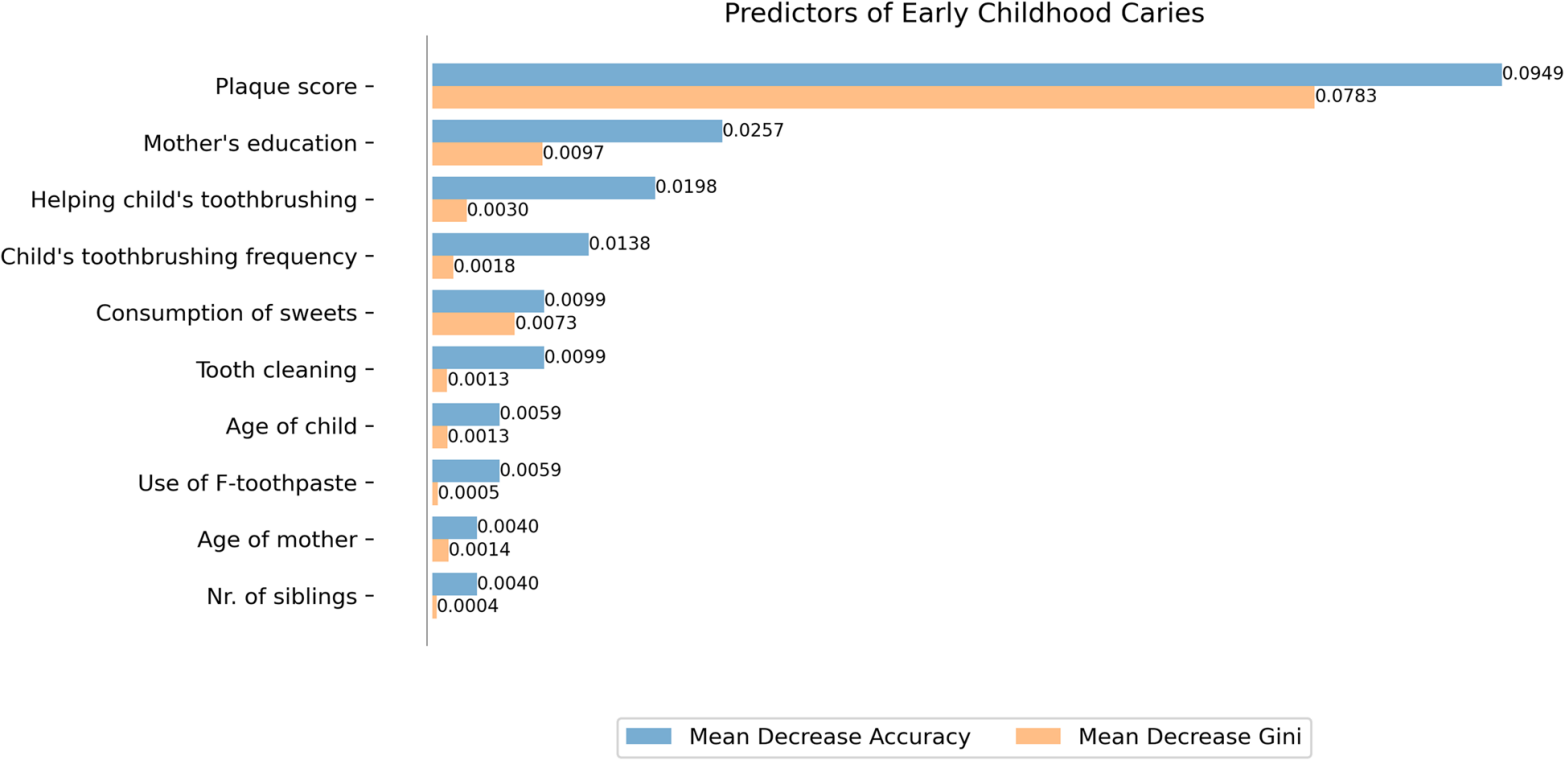
Demographic and oral health related predictors of ECC with MDG and MDA measures, where higher MDG and MDA values indicate greater variable importance

Figure 3 shows the order of importance feature classifying ECC according to MDG and MDA. Features of the model’s MDG and MDA are the following: plaque score (MDG: 0.08, MDA: 0.10), mother’s education (MDG: 0.01, MDA: 0.025), child’s toothbrushing frequency (MDG: 0.003, MDA: 0.02).

**Fig. 4:**
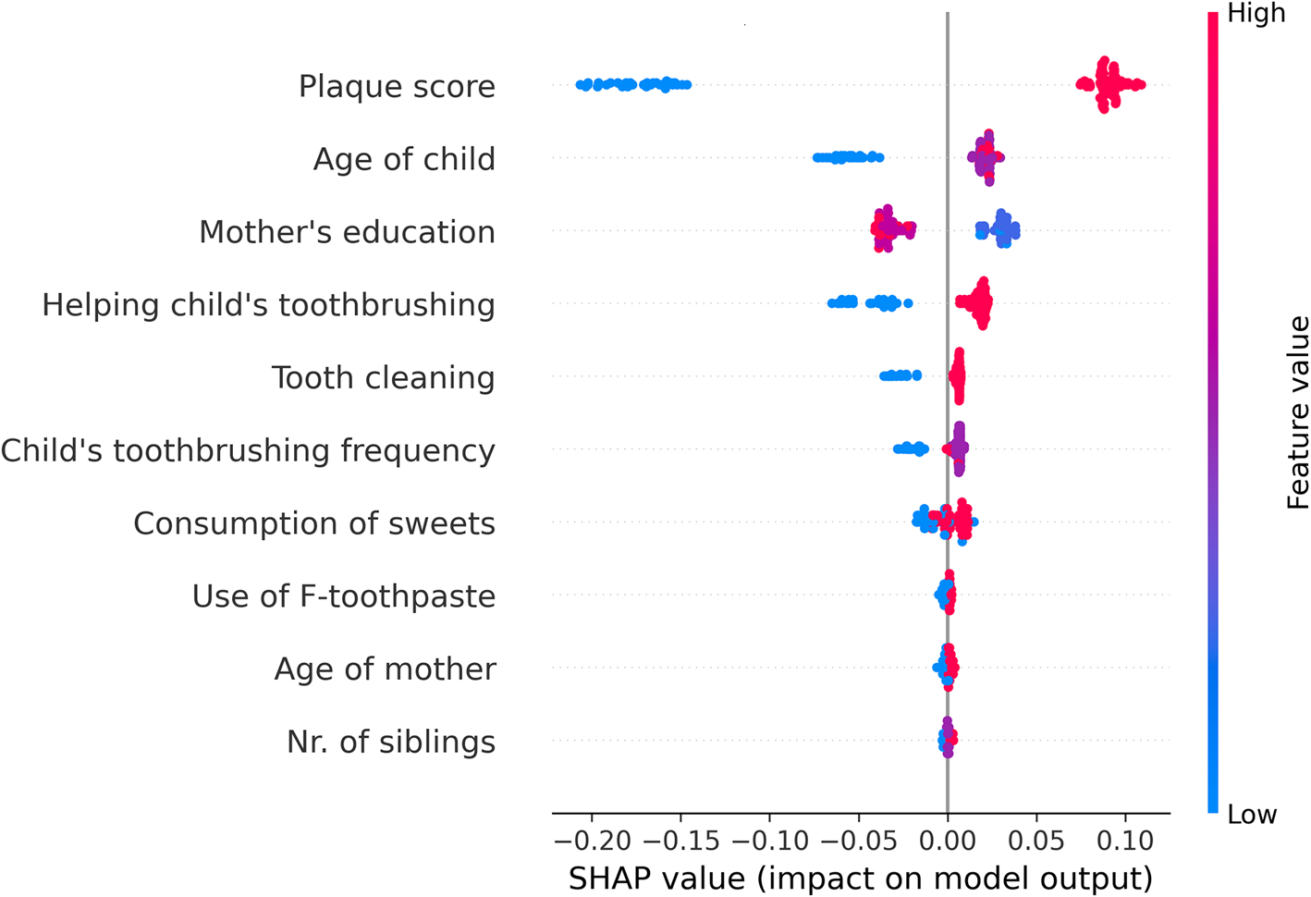
SHAP summary plot illustrates feature impact on ECC predictions of the global interpretability of the Model, each point represents a specific instance, colour code of feature values indicate the contribution to the model outcome (ECC)

Figure 4 illustrates the SHAP global plot, which represents the directional impact of different features on the model’s predictions. The prevalence of ECC was higher with a higher plaque score. Mothers with lower education (basic or primary) had children with higher prevalence of ECC. The prevalence of ECC was higher in older children although this feature had less importance than the plaque score. Features such as number of siblings, age of mother and use of fluoridated toothpaste had minimal impact on prediction. Mothers who did not help their child during brushing increased possibility of having ECC.

## Discussion

Our developed ML model showed promising ability to classify children based on their ECC risk status and thus facilitate informed decision-making for prevention and therapeutic care. The ML model identified 10 features as the most relevant for ECC prediction. The ML algorithm with the RFC model together with RFE feature selection, demonstrated the highest and consistent performance. The SHAP method enabled a deeper understanding of the model and the impact and direction of features on prediction. The new approach enhances interpretability, simplifying the identification of predictors and supporting subsequent decision-making processes [43]. The present study has implications for the use of ML in settings with insufficient number of dental care providers leading to expensive dental care, especially in the low- and middle-income countries.

Dental plaque score ranked top in our ML model as reported by others [44] and confirming our previous findings using traditional regression modelling on the same dataset [17, 31]. In addition, like previous research, this study shows that the higher the age of the child, the higher the risk of ECC [17, 31], and the higher the age of the mother and lower the level of education, the higher the possibility of ECC [27, 45]. Although mother’s age had the minimal impact on the model, we included it to enhance the accuracy.

In addition, the predicting oral health behaviour variables associated with ECC - lower frequency of child’s toothbrushing, limited use of fluoridated toothpaste, and frequent consumption of sweets [27, 45–47] - were included in the final model and demonstrated to be associated with ECC unlike what we found in the traditional regression analysis [17, 31]. Furthermore, we identified that lack of assistance in child’s toothbrushing and limited use of appropriate tooth cleaning means (toothbrush and toothpaste) as important predicting factors for ECC like in previous studies [48, 49] but not with our traditional regression analysis [17, 31]. Interestingly, the model included the number of siblings as predictor of ECC in this population although the feature itself had the least importance.

In this study, several machine learning algorithms including RFC, XGBoost, AdaBoost, SVM, and MLP were used to build a predictive model for ECC. Of these methods, RFC together with RFE feature selection (10 features) demonstrated the highest performance of the test set with the AUC-ROC score 0.77 and accuracy 0.72. The parameters of this model are in line with some previous studies. For example, Park et al. reported that the RFC model predicted ECC in Korean children achieving an AUC value of 0.780 [27] and an American study reported an AUC of 0.74 and sensitivity of 0.67 [28]. While Ramos-Gomez et al. found an accuracy of 0.71 in their validation set [40], the accuracy of our validation set was 0.76. The performance of our model underscores its efficacy in predicting ECC and demonstrates its suitability for practical application in this field.

One of the strengths of our study is the integration of both survey and clinical data from a representative sample, offering a comprehensive view of caries risk factors in the Bangladeshi child population. Developing individual prediction models tailored for specific populations is crucial because they account for variations in risk factors and oral health behaviours unique to each demographic group [50]. By accurately capturing these distinctions, tailored models ensure risk quantification and enable targeted preventive interventions that are culturally and contextually relevant, ultimately enhancing the effectiveness of oral health initiatives within a population based on a precision public health approach [22].

However, the study has some limitations. One limitation was the sample size used for ML modelling: larger samples potentially enhance accuracy while smaller datasets may limit the effectiveness of ML to predict caries. Another methodological limitation is that we used target encoding, which may lead to information leakage, resulting in overfitting. Our findings are relevant to Bangladeshi children, and one should be cautious to generalize these to other populations. Incorporating additional variables such as socioeconomic background, genetic predispositions, oral microbiome composition, malocclusion, and systemic health conditions could potentially enhance the predictive modelling outcomes [45, 46, 51]. Even though our data was primarily cross sectional, it covers a longitudinal span of child age, allowing for insights into ECC development over time. Nevertheless, to maximize the efficacy of ML in predicting ECC, future studies should explore using longitudinal data.

Despite the limitations, our proposed ML model makes a significant contribution to the field by predicting ECC using survey and clinical examination data. By analysing large datasets and identifying patterns and risk factors, ML models can predict ECC in child populations thereby helping with the planning of interventions. The use of ML in dental caries research can benefit public dental care, enabling policymakers to make informed decisions about personalized prevention strategies and interventions, leading to improved oral health outcomes. Our findings demonstrate the feasibility and usefulness of using this approach to support the analysis of survey data to identify ECC risk patterns in a limited-resources setting like Bangladesh.

## Conclusion

The developed new ML model to predict ECC in Bangladeshi children under five years used 10 critical health behaviours of mother-child pairs to predict ECC experience with good accuracy. ECC risk indicators identified were Plaque score, child’s age, mother’s age, maternal education, number of siblings, assistance with brushing, tooth cleaning, brushing frequency, sweets consumption, use of fluoride toothpaste. The model presents an option to allow ECC prediction for targeted preventive programs in a low resource setting. Further research and validation are recommended to optimize the accuracy and reliability of the predictive model in other target groups and settings.

## Data Availability

All data and materials supporting the findings of this study are included in the article and its Supplementary Material. For further inquiries regarding the data, contact the corresponding author.

## Abbreviations

ECC: Early childhood caries
ML: Machine Learning
AI: Artificial intelligence
RFE: Recursive feature elimination
RF: Random forest
RFC: Random forest classifier/ classification
XGBoost: Extreme gradient boosting
SVM: Support vector machine
AdaBoost: Adaptive boosting
MLP: Multi-layer perceptron
SHAP: Shapley Additive Explanations
MDG: Mean decrease gini
MDA: Mean decrease accuracy
ROC: Receiver operating characteristic
AUC-ROC: Area under the ROC curve
ICDAS II: International caries detection and assessment system II
GridSearchCV: Grid search cross validation
K-fold CV: K-fold cross-validation
